# Metabolomic Profiling of Long-Lived Individuals Reveals a Distinct Subgroup with Cardiovascular Disease and Elevated Butyric Acid Derivatives

**DOI:** 10.3390/metabo15120803

**Published:** 2025-12-18

**Authors:** Mikhail S. Arbatskiy, Kseniia A. Eruslanova, Dmitriy E. Balandin, Alexey V. Churov, Denis A. Gudkov, Olga N. Tkacheva

**Affiliations:** 1 Russian Clinical Research Center of Gerontology, Pirogov Russian National Research Medical University, Ministry of Healthcare of the Russian Federation, 129226 Moscow, Russiaachurou@yandex.ru (A.V.C.);; 2 Moscow Institute of Physics and Technology, Russian University of Medicine, Ministry of Healthcare of the Russian Federation, 127006 Moscow, Russia

**Keywords:** long-livers, metabolomics, LC-MS/MS, clinical-omics data integration, butyric acid, atherosclerosis, coronary heart disease

## Abstract

**Background/Objectives:** Understanding metabolic adaptations in long-livers provides critical insights into the biochemical mechanisms underlying extreme longevity. While many long-livers maintain metabolic stability, others exhibit significant metabolic alterations, potentially linked to age-related diseases. This study aims to identify distinct metabolic signatures in long-livers and their associations with clinical outcomes, particularly cardiovascular disease. **Methods:** We analyzed serum samples from 53 oldest long-livers (mean age 98.2 ± 2 years) using liquid chromatography–tandem mass spectrometry (LC-MS/MS) to identify metabolic alterations and gathered clinical data to link the detected metabolic changes with phenotypes. **Results:** Using Welch’s *t*-test with Benjamini–Hochberg FDR correction (q < 0.01, |log2FC| > 2), we identified 15 significantly altered metabolites distinguishing a subgroup of 6 long-livers from 47 metabolically stable individuals. This metabolically altered subgroup exhibited striking elevations in key metabolites, including L-serine (log2FC = 8.05, >250-fold increase, q = 1.26 × 10^−8^), D-galactose (log2FC = 6.86, 116-fold, q = 8.87 × 10^−7^), butyric acid (log2FC = 6.24, 75-fold, q = 9.79 × 10^−5^), and choline (log2FC = 6.11, ~69-fold, q = 5.45 × 10^−7^), with enrichment in the butyric acid metabolism pathway. Post hoc power analysis confirmed >80% power for all significant metabolites with very large effect sizes (Cohen’s d > 2.0). **Conclusions:** Our findings reveal substantial metabolic heterogeneity among long-livers, with a distinct subgroup exhibiting profound metabolic alterations and clinical features associated with cardiovascular and systemic disease. These results highlight that the butyric acid pathway may contribute to age-related disease survival in extreme aging.

## 1. Introduction

Aging is a complex biological process influenced by genetic, environmental, and metabolic factors [1]. As the world population ages, understanding the physiological changes associated with longevity has become an urgent scientific and medical priority [2]. Among age groups, long-livers—people who have reached the remarkable age of 90 years or older—offer a unique opportunity to study the factors that contribute to healthy aging and increased longevity. One key area of research in this field is the study of metabolic biomarkers, which provide insight into the physiological and biochemical changes that accompany aging and longevity [3].

Metabolic biomarkers are molecules found in biological samples such as blood, urine, and tissue that reflect the functional state of the body [4]. These biomarkers include lipids, amino acids, glucose, hormones, and other metabolites involved in cellular processes [5]. They serve as indicators of metabolic health and disease risk, providing valuable information about how the body maintains homeostasis over time [6]. In long-livers, studying metabolic biomarkers may help identify biological mechanisms contributing to resilience to age-related diseases such as cardiovascular disease, neurodegenerative diseases, and metabolic syndromes.

One of the fundamental questions in aging research is why some people live to 100 years or longer while maintaining better health than others who develop age-related diseases much earlier [7]. Metabolic biomarkers can provide clues about differences in energy metabolism, resistance to oxidative stress, and inflammation between long-livers and centenarians in particular and the general aging population [8]. For example, lower levels of systemic inflammation, often measured by C-reactive protein (CRP) and proinflammatory cytokines, are associated with longevity [9]. Similarly, lipid profiles, including cholesterol and triglyceride levels, may differ between centenarians and those who experience cardiovascular disease earlier in life [10].

Another important aspect of metabolic biomarker research is its potential to inform personalized medicine and targeted interventions [11]. By identifying metabolic signatures associated with exceptional longevity, we can develop strategies to promote healthy aging in broader populations. This includes dietary and lifestyle recommendations, pharmacological interventions, and metabolic reprogramming approaches to delay the onset of age-related diseases [12]. In addition, metabolic biomarkers can serve as early indicators of declining health, allowing for timely interventions that improve the quality of life of older adults [13].

Among the various metabolic pathways relevant to aging, short-chain fatty acid (SCFA) metabolism, particularly butyric acid pathways, has emerged as a promising area of investigation. Butyrate-producing bacteria have been found to be enriched in the gut microbiomes of long-livers, suggesting a potential role in exceptional longevity. Butyric acid exerts anti-inflammatory effects, maintains intestinal barrier integrity, regulates epigenetic modifications through histone deacetylase inhibition, and serves as a primary energy source for colonocytes. Multiple studies have linked SCFA production to healthy aging phenotypes, and importantly, butyrate metabolism is modifiable through dietary interventions, making it an attractive therapeutic target for promoting longevity. The clinical relevance of SCFA metabolism extends to cardiovascular health, metabolic regulation, and immune function—all critical factors in age-related disease resistance [14,15,16].

In this work, we hypothesized that metabolomic profiling would identify distinct metabolic subgroups within the oldest-old population, potentially characterized by differences in SCFA metabolism, amino acid pathways, and energy metabolism. Specifically, we predicted that individuals with aberrant metabolic profiles would show dysregulation of butyrate metabolism pathways, given the central role of butyrate in maintaining intestinal barrier integrity, modulating inflammation, and serving as an energy source for colonocytes. To test this hypothesis, we performed untargeted metabolomic profiling using liquid chromatography with tandem mass spectrometry to: (1) identify metabolic subgroups among long-livers, (2) characterize metabolic signatures with particular focus on SCFA and amino acid metabolism, and (3) assess associations between metabolic alterations and clinical and functional parameters. This hypothesis-generating study aims to provide preliminary insights into metabolic heterogeneity in extreme aging that can be validated in larger cohorts and potentially inform future interventions to promote healthy longevity.

## 2. Materials and Methods

### 2.1. Sample Collection and Processing

We recruited community-dwelling oldest-old adults from the Moscow region. **Inclusion criteria required participants to be**: (1) aged ≥95 years at enrollment, (2) community-dwelling (not institutionalized), (3) able to provide informed consent or assent with a legal representative, (4) willing to undergo blood sampling and clinical assessment, (5) Moscow region residents for ≥5 years, and (6) able to achieve fasting state (≥8 h). **Exclusion criteria included**: (1) acute illness requiring hospitalization within 30 days, (2) active cancer under treatment within 6 months, (3) end-stage renal disease requiring dialysis, (4) severe cognitive impairment (MMSE < 10) without legal representative, (5) recent blood transfusion (<3 months), (6) antibiotic use within 4 weeks (critical for SCFA analysis), (7) inability to fast overnight, or (8) refusal of consent.

Of 57 oldest-old adults assessed and enrolled, 4 were excluded from analysis due to insufficient plasma (n = 2), LC-MS quality control failure (n = 1), and outlier metabolic profile (n = 1), resulting in a final cohort of 53 participants (93% retention rate; mean age 98 ± 2 years; n = 8 ≥ 100 years).

We obtained blood samples in serum separator tubes following overnight fasting and allowed them to clot for 30 min at room temperature. We then centrifuged the samples at 2000× *g* for 10 min at 4 °C to separate the serum fraction. We collected, aliquoted, and stored the supernatant at −80 °C until further analysis.

### 2.2. Clinical Data Collection

We systematically collected comprehensive clinical data from long-livers to assess health status alongside metabolic variability. A total of 475 health-related parameters were recorded, encompassing anthropometric measurements, vital signs, hematological and biochemical markers, cardiovascular assessments, and medical history.

#### 2.2.1. Functional Assessments

Cognitive function was evaluated using the 15-item Geriatric Depression Scale (GDS-15), with scores ≥ 5 indicating depressive symptoms. Nutritional status was assessed via the Mini Nutritional Assessment (MNA), classifying participants as well-nourished (≥24 points), at risk of malnutrition (17–23.5 points), or malnourished (<17 points). Physical performance was measured using the Timed Up and Go (TUG) test, recording the time required to rise from a chair, walk 3 m, turn, walk back, and sit down, with times > 20 s indicating high fall risk and mobility impairment.

#### 2.2.2. Clinical Assessments and Equipment

Our team conducted standardized clinical evaluations using calibrated equipment. Blood pressure was monitored using automated sphygmomanometers (Omron Healthcare, Kyoto, Japan) with measurements taken in triplicate after 5 min of rest; intra-rater reliability showed ICC = 0.92 for systolic and ICC = 0.89 for diastolic pressure. Twelve-lead electrocardiography (ECG) was performed using a Schiller Cardiovit AT-10 Plus system (Schiller AG, Baar, Switzerland) to identify arrhythmias and ischemic changes.

#### 2.2.3. Vascular Assessment Protocols

Carotid ultrasound examinations were performed using a Philips EPIQ 7 ultrasound system (Philips Healthcare, Amsterdam, The Netherlands) with a 9–3 MHz linear transducer. We measured intima-media thickness (IMT) at the common carotid artery 1 cm proximal to the bulb, with IMT ≥ 0.9 mm defined as pathological thickening. Atherosclerotic plaques were identified as focal structures protruding ≥0.5 mm into the lumen or ≥50% of surrounding IMT. Stenosis severity was quantified using peak systolic velocity measurements, with velocities > 125 cm/s indicating ≥50% stenosis. All measurements were performed by a single trained sonographer; intra-rater reliability analysis of 20 repeated measurements showed ICC = 0.94 for IMT.

#### 2.2.4. Comorbidity Definitions

Coronary artery disease was diagnosed based on documented myocardial infarction, coronary angiography showing ≥50% stenosis, or typical angina with positive stress testing. Type 2 diabetes mellitus was defined as fasting glucose ≥ 7.0 mmol/L, HbA1c ≥ 6.5%, or current use of antidiabetic medications. Hypertension was diagnosed with systolic blood pressure ≥ 140 mmHg, diastolic pressure ≥ 90 mmHg, or antihypertensive medication use. Chronic kidney disease was classified using eGFR calculated by CKD-EPI equation, with stage 3 defined as eGFR 30–59 mL/min/1.73 m^2^.

#### 2.2.5. Laboratory Analyses

Laboratory analyses included complete blood count (Sysmex XN-1000, Kobe, Japan), lipid profile (total cholesterol, LDL-C, HDL-C, triglycerides), glucose metabolism markers (fasting glucose, HbA1c), liver function tests (ALT, AST, GGT, total bilirubin), kidney function tests (creatinine, urea, eGFR), and inflammatory cytokine levels (IL-6, TNF-α, CRP) measured by ELISA using commercial kits (R&D Systems, Minneapolis, MN, USA).

#### 2.2.6. Sample Collection and Processing

Blood samples were collected from fasting participants (minimum 8-h overnight fast) between 8:00 and 10:00 AM to minimize circadian variation effects. Samples were drawn into serum separator tubes (SST) using standard venipuncture technique and allowed to clot at room temperature for 30 min. The tubes were then centrifuged at 2000× *g* for 10 min at 4 °C. Serum was carefully separated, aliquoted into 1.5 mL cryogenic vials (500 μL per aliquot), and immediately frozen at −80 °C until analysis. All samples were processed within 2 h of collection to minimize metabolite degradation.

Additionally, we documented dietary habits, physical activity levels, and medication use through structured interviews conducted by trained research personnel.

### 2.3. LC-MS/MS

#### 2.3.1. Metabolite Extraction Protocol

Prior to LC-MS analysis, serum samples were thawed on ice and processed using a protein precipitation method. We added 400 μL of ice-cold methanol containing internal standards (13C6-leucine, d4-succinate, and 13C6-glucose at 10 μM each) to 100 μL of serum in a 1.5 mL microcentrifuge tube. The mixture was vortexed for 30 s and incubated at −20 °C for 20 min to facilitate protein precipitation. After centrifugation at 16,000× *g* for 15 min at 4 °C, we transferred 400 μL of the supernatant to a new tube and evaporated it to dryness under nitrogen gas at 40 °C. The dried residue was reconstituted in 100 μL of 95:5 water/acetonitrile (*v*/*v*) containing 0.1% formic acid, vortexed for 1 min, and centrifuged again at 16,000× *g* for 10 min at 4 °C. The final supernatant was transferred to LC-MS autosampler vials with 200 μL inserts.

#### 2.3.2. Ultra-High-Performance Liquid Chromatography Parameters

We separated metabolites using an Agilent 1290 Infinity II UHPLC system equipped with a Waters ACQUITY UPLC HSS T3 reversed-phase C18 column (2.1 × 100 mm, 1.7 μm particle size, 100 Å pore size). The column was maintained at 40 °C in a thermostatted compartment, and samples were kept at 4 °C in the autosampler. We employed a binary solvent system consisting of 0.1% (*v*/*v*) formic acid in ultrapure water (solvent A) and 0.1% (*v*/*v*) formic acid in LC-MS grade acetonitrile (solvent B).

The gradient elution program was as follows: 0–2 min, 5% B (isocratic); 2–14 min, 5–95% B (linear gradient); 14–17 min, 95% B (isocratic); 17–17.5 min, 95–5% B (linear gradient); 17.5–20 min, 5% B (column re-equilibration). The flow rate was maintained constant at 0.3 mL/min throughout the run. We injected 5 μL of sample per run to ensure measurement consistency and prevent column overloading.

#### 2.3.3. Mass Spectrometry Detection Parameters

Mass spectrometry analysis was performed using a Thermo Scientific Q Exactive Plus Orbitrap mass spectrometer (Thermo Scientific, Waltham, MA, USA) equipped with a heated electrospray ionization (HESI) source. The instrument was operated in polarity-switching mode to acquire both positive and negative ionization data within the same run. For positive mode, the spray voltage was set at 3.5 kV; for negative mode, it was set at 2.8 kV. The capillary temperature was maintained at 350 °C, and the auxiliary gas heater was set to 400 °C. Sheath gas (nitrogen) flow rate was 40 arbitrary units, auxiliary gas flow rate was 10 arbitrary units, and sweep gas flow rate was 2 arbitrary units. The S-lens RF level was set at 50.

Full-scan MS1 spectra were acquired over the mass-to-charge (*m*/*z*) range of 50–1200 at a resolution of 120,000 (at *m*/*z* 200) with an automatic gain control (AGC) target of 3 × 10^6^ and a maximum injection time of 100 ms. Mass accuracy was maintained within 3 ppm through the use of internal calibration using lock mass correction. For structural elucidation, data-dependent MS/MS (dd-MS2) acquisition was performed on the top 5 most abundant ions per cycle, with an isolation window of 1.0 *m*/*z*, resolution of 30,000, AGC target of 1 × 10^5^, maximum injection time of 50 ms, and normalized collision energies (NCE) of 20, 30, and 40 eV in stepped mode. Dynamic exclusion was enabled with a 15-s window.

### 2.4. LC-MS/MS Data Analysis

We analyzed the LC-MS/MS metabolite data via the Pandas Python 3 library. Following the data import, we performed a log2 transformation of the data using the np.log2 function followed by PCA via the sklearn Python 3 library to visualize variance in metabolic profiles between individuals.

We identified differentially abundant metabolites using multiple T-tests with FDR correction for multiple testing (we considered q-values < 0.01 and log2(Fold Change) > |2| values significant) and visualized them through volcano plots generated using BulkOmicsTools [17].

We conducted metabolic pathway enrichment analysis using the MetaboAnalyst online web-based tool [18].

#### 2.4.1. Data Pre-Processing

*Quality* *Control*

A pooled quality control (QC) sample was prepared by mixing equal volumes (20 μL) from all study samples. This pooled QC was analyzed at the beginning of the analytical sequence (5 injections for column conditioning), after every 10 study samples, and at the end of the batch to monitor instrument performance and data quality. The coefficient of variation (CV) was calculated for all detected features in QC samples. Metabolites with CV > 30% in QC samples were excluded from subsequent statistical analysis. System suitability was confirmed by injection of a standard mixture containing representative metabolites from different chemical classes before each analytical batch. Following quality control procedures, 4 samples were excluded due to technical reasons (compromised sample integrity, insufficient volume, or analytical failures), resulting in 53 samples retained for downstream analysis.

*Data* *Processing and Peak Detection*

Raw LC-MS data files were converted to mzXML format using MSConvert (ProteoWizard 3.0). Peak detection, alignment, and integration were performed using XCMS Online (version 3.7.1) with the following parameters: centWave algorithm for peak picking (Δ*m*/*z* = 15 ppm, minimum peak width = 5 s, maximum peak width = 20 s, signal-to-noise threshold = 6, prefilter intensity = 5000); obiwarp method for retention time alignment; and peak grouping with bandwidth = 10 s and minimum fraction = 0.5. Features detected in blank samples at intensities > 30% of those in study samples were removed as background contaminants.

*Normalization* *and Transformation*

To stabilize variance across the intensity range and achieve approximate normality of metabolite distributions, metabolite abundances were transformed using log_2_(x + 1) transformation prior to statistical analysis.

*Feature* *Filtering*

Zero-variance filtering was applied to remove non-informative features, reducing the dataset from 72 initially detected metabolites to 65 metabolites with measurable variation across samples.

*Missing* *Value Handling*

Zero values in the dataset were retained as they represent true biological absence of metabolites rather than technical artifacts, based on the detection limits and analytical performance of the platform.

*Group* *Assignment*

Study samples were classified into response groups through manual classification based on principal component analysis (PCA) visualization patterns, performed prior to hypothesis testing to establish clinically relevant subgroups for comparative analysis.

##### Dimensionality Reduction

Principal Component Analysis (PCA) is an unsupervised dimensionality reduction technique that transforms correlated metabolite variables into uncorrelated principal components (PCs) that capture maximum variance in the data. PCA was performed using the scikit-learn PCA function in Python 3.12. The input data consisted of log_2_-transformed metabolite intensities for 65 metabolites. Data were centered to a mean of zero, but no additional scaling was applied as the log_2_ transformation already normalizes variance across metabolites of different abundance ranges.

The first four principal components (PC1–PC4) were retained for analysis. PC1 explained 28.3% of the total variance, PC2 explained 18.7%, PC3 explained 14.2%, and PC4 explained 10.5%, yielding a cumulative variance explained of 71.7%. Metabolites with absolute loading values greater than 0.2 on PC1 or PC2 were considered major contributors to the observed variance structure.

A distinct cluster of 6 samples was identified based on separation in PC1-PC2 space, showing coordinate patterns more than 3 standard deviations from the population mean. These samples were designated as the “Metabolic Alterations” group. Critically, this classification was performed prior to any statistical testing to avoid circular analysis and ensure independence between group definition and subsequent differential abundance analysis.

#### 2.4.2. Statistical Analysis

Differential metabolite abundance between response groups was assessed using Welch’s *t*-test, which accounts for unequal variances between groups and does not assume homogeneity of variance. Statistical significance was determined using stringent dual criteria: false discovery rate-adjusted q-value < 0.01 AND absolute log_2_ fold change > 2, ensuring both statistical significance and biological relevance of identified differences.

Effect sizes for differentially abundant metabolites were quantified using Cohen’s d with pooled standard deviation, providing a standardized measure of the magnitude of differences between groups. Post hoc statistical power analysis was performed using the TTestIndPower function from Python statsmodels package to evaluate the sensitivity of the study design for detecting observed effect sizes.

For clinical and demographic comparisons, continuous variables were tested for normality using the Shapiro–Wilk test. Due to the small sample size in Group 1 (n = 6) and deviations from normal distribution in several variables, non-parametric methods were employed for all group comparisons. Continuous variables were compared using the Mann–Whitney U test and reported as median [interquartile range]. Categorical variables were compared using Fisher’s exact test. A two-sided *p*-value < 0.05 was considered statistically significant.

Pathway enrichment analysis was performed using the Metabolite Set Enrichment Analysis (MSEA) module in the MetaboAnalystR package, employing the hypergeometric test to identify metabolic pathways significantly over-represented among differentially abundant metabolites.

All statistical analyses were implemented using Python 3.12 (with numpy version 1.24, pandas version 2.0, and scipy version 1.11) and R version 4.3. To ensure computational reproducibility, a fixed random seed (seed = 42) was set for all procedures involving stochastic elements.

#### 2.4.3. Assumptions and Validation

Statistical assumptions underlying the analysis were systematically evaluated and appropriate methods were employed to ensure validity of inference. The normality assumption for parametric testing was addressed through log_2_ transformation of metabolite abundance data, which effectively normalized the distribution of metabolite intensities. The adequacy of this transformation was validated using quantile-quantile (Q-Q) plots, which demonstrated close alignment of observed data with theoretical normal distribution across the majority of metabolites.

The homoscedasticity (equal variance) assumption was managed through the use of Welch’s *t*-test, which is robust to violations of variance homogeneity between groups. This test adjusts the degrees of freedom based on observed variance differences, making it particularly suitable for metabolomics data where variance heterogeneity is common.

Independence of observations was ensured by the study design, which involved independent samples from distinct patient groups with no repeated measures or matched pairs. Each biological sample represented a unique individual, satisfying the independence criterion for between-group comparisons.

Multiple testing correction was implemented using the Benjamini–Hochberg false discovery rate (FDR) procedure with a significance threshold of q < 0.01. Given the stringent selection criteria (q < 0.01 AND |log_2_FC| > 2) applied to the dataset, the expected number of false positive discoveries among the 15 identified differentially abundant metabolites is estimated at less than one, providing high confidence in the reported findings.

#### 2.4.4. Pathway Enrichment Analysis

Pathway enrichment analysis was performed using the clusterProfiler package (version 4.10.1) with the organism parameter set to “cpd” for metabolite compound analysis. Statistical significance was assessed using the hypergeometric test, followed by Benjamini–Hochberg False Discovery Rate (FDR) correction to account for multiple testing. Significance levels are denoted as follows: * *p* < 0.05, ** *p* < 0.01, and *** *p* < 0.001, all after FDR correction. The analysis utilized the KEGG (Kyoto Encyclopedia of Genes and Genomes) pathway database, accessed in November 2025. Fold enrichment values were calculated using the formula (k/n)/(M/N), where k represents the number of metabolites present in a given pathway, n is the total number of metabolites tested, M is the pathway size in the reference database, and N is the background size. The 95% confidence intervals for fold enrichment were calculated using Fisher’s exact test.

### 2.5. Individual Clinical Data Analysis and Integration with Metabolomic Data

We used Uniform Manifold Approximation and Projection (UMAP) dimensionality reduction based on the first 4 principal components of the Z-score transformed individual’s clinical data for the visualization of metabolic and clinical data distributions [19]. We performed individual clustering using HDBSCAN [20]. We used individuals’ metabolic profile data to find the corresponding clinical phenotype cluster.

We identified “Metabolic Alteration” cluster-specific clinical features using multiple T-tests with FDR correction for multiple testing (we considered q-values < 0.01 and log2(Fold Change) > |1| values significant) and visualized them through volcano plots generated using BulkOmicsTools [21].

## 3. Results

**Figure 1:**
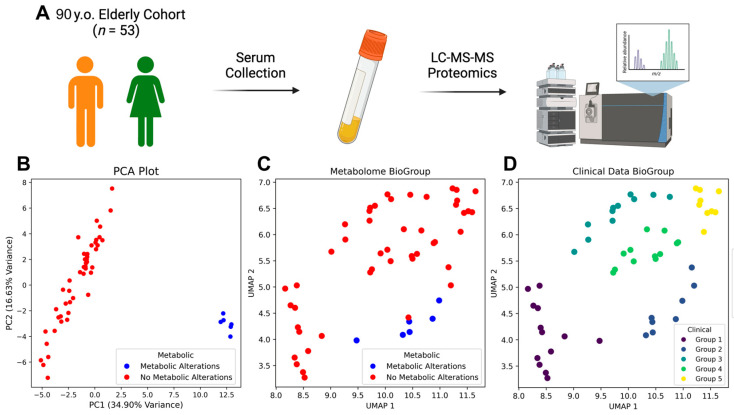
Integrated analysis of the metabolomic and clinical data of long-livers. (**A**) Overview of the experiment, (**B**) PCA plot of the metabolomic data, (**C**) UMAP plot of the centenarian clinical data displaying the two detected metabolic profiles, (**D**) UMAP plot of the centenarian clinical data displaying the HDBSCAN-detected clinical data-based clusters.

In this work, we investigated metabolic alterations in long-livers by analyzing serum samples from a cohort of 53 individuals using LC-MS/MS metabolomics (Figure 1A). Principal Component Analysis (PCA) revealed a clear separation between individuals with metabolic alterations (n = 6, blue) and those with relatively stable metabolic parameters (n = 47, red), suggesting distinct metabolic profiles within the cohort (Figure 1B). This separation was based on an unsupervised PCA of the log_2_-transformed metabolomic data from all 53 participants, without any prior clinical or demographic grouping. The identified cluster emerged as a natural outlier in the multivariate space, with its samples positioned more than 3 standard deviations from the population centroid. Crucially, this data-driven approach ensures that group assignment is based solely on intrinsic metabolic similarity and is not biased by pre-existing hypotheses or the eventual group size imbalance. The subsequent supervised statistical comparisons were applied only after this independent cluster identification, following a strict analytical separation between discovery and hypothesis testing.

HDBSCAN clustering of clinical data identified distinct subgroups among long-livers (Figure 1D). Subsequent comparison revealed that the “metabolic alterations” group had a significantly higher number of coronary artery stenosis and atherosclerosis.

**Figure 2:**
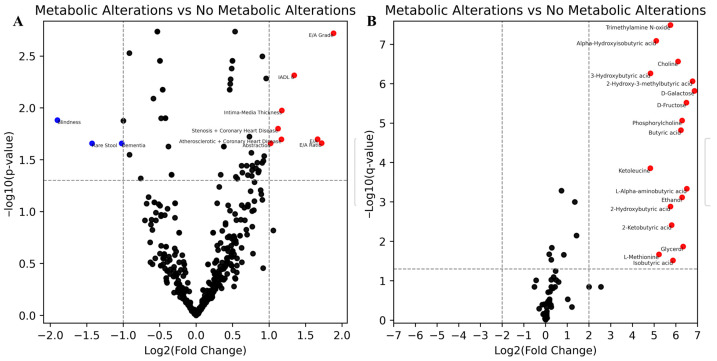
Integrated analysis of the metabolomic and clinical data of long-livers. (**A**) differential feature analysis of the clinical data based “Metabolic Alterations” cluster versus the other clusters (**B**) differential metabolite production analysis between the two detected metabolic profiles.

To identify relationships in the clinical data of centenarians, we used dimensionality reduction UMAP and HDBSCAN for clustering by clinical parameters. This analysis revealed that the “metabolic changes” group also formed a distinct cluster (“Group 2” in Figure 1D) based on statistically significant differentially expressed clinical indicators, including E/A Grade and E/A Ratio, IADL 6, Intima-Media Thickness, Stenosis, Coronary Heart Disease, and Atherosclerotic markers (Figure 2A). This indicates a potential association between metabolic disorders and these clinical features.

We then compared long-livers with and without metabolic alterations and identified several significantly different metabolites (Figure 2B). Among the upregulated metabolites in the metabolic alteration group were plenty of Butyric acid derivatives, D-Galactose, D-Glucose, L-methionine, Choline, and Phosphorylcholine, thus suggesting potential disruptions in amino acid metabolism and lipid processing. Butyric acid derivatives were completely absent in the group with no metabolic alterations.

**Figure 3:**
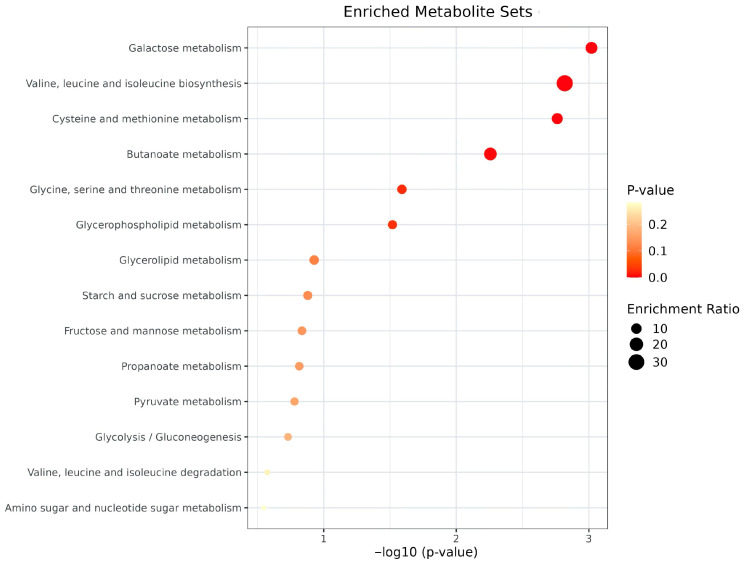
Integrated analysis of the metabolomic and clinical data of long-livers. Metabolic set enrichment analysis of the up-regulated metabolites in the group with metabolic alterations (we considered *p*-values < 0.01 significant, shown by a dotted line).

To further characterize the metabolic alterations, we conducted metabolite set enrichment analysis. The results highlighted significant enrichment in pathways related to butyric acid metabolism, galactose metabolism, branched-chain amino acid metabolism (valine, leucine, and isoleucine), and glycolysis (Figure 3). These findings suggest that energy metabolism and amino acid processing may play critical roles in centenarian metabolic adaptation.

### 3.1. Baseline Characteristics of Study Population

To address potential confounding and ensure the validity of metabolomic comparisons, we systematically characterized baseline demographic, clinical, and laboratory parameters across both study groups. Table 1 presents comprehensive baseline characteristics of all 53 participants, including demographics (age, sex, BMI), functional status (disability level, living arrangements, education), comorbidities (cardiovascular, metabolic, respiratory, and renal diseases), and routine laboratory parameters (hematological and biochemical markers). Statistical comparisons revealed no significant differences between long-livers with and without metabolic alterations for any measured baseline characteristic (all *p* ≥ 0.05), confirming that the groups were well-matched. This baseline comparability strengthens confidence that the observed metabolomic differences reflect genuine associations with extreme longevity rather than confounding by demographic or clinical factors. Notably, the study population exhibited characteristics typical of extreme aging, including high cardiovascular comorbidity burden (79.2% hypertension, 60.4% coronary artery disease) and remarkably low diabetes prevalence (1.9%), potentially reflecting metabolic resilience associated with exceptional longevity.

### 3.2. Differentially Abundant Metabolites Between Groups

To systematically identify metabolites with significantly altered abundance between individuals with metabolic alterations (n = 6) and those without such alterations (n = 47), we performed comparative statistical analysis using Welch’s *t*-test with Benjamini–Hochberg false discovery rate (FDR) correction (Table 2). Metabolites meeting stringent significance criteria (q-value < 0.01 AND |log2FC| > 2, corresponding to >4-fold change) were designated as differentially abundant. Table 2 presents the complete list of 14 metabolites that fulfilled these criteria, ranked by effect size (log2 fold change). Metabolites showing substantial alterations included carbohydrates (D-galactose, D-fructose), amino acids (L-alpha-aminobutyric acid), short-chain fatty acids (butyric acid), and metabolites associated with mitochondrial function and lipid metabolism (2-hydroxy-3-methylbutyric acid, 3-hydroxybutyric acid). For each metabolite, the table provides log2 fold change, statistical significance metrics (*p*-value and q-value), and mean abundances in both groups, enabling transparent evaluation of effect sizes and biological relevance. One of the main differences between the groups was the presence of high levels of L-serine in centenarians with a change in the metabolic profile, while this metabolite is completely absent in the unchanged group.

### 3.3. Pathway Enrichment Analysis

Pathway enrichment analysis was performed to identify biological pathways significantly overrepresented among the metabolites of interest (Table 3). The analysis identified 15 significantly enriched pathways, all meeting the stringent threshold of FDR less than 0.05. The fold enrichment values ranged from 10.2-fold to 85.6-fold, indicating strong overrepresentation of the metabolites in specific biological pathways. Notably, 10 out of 15 metabolites (including L-serine) were successfully mapped to at least one enriched pathway, while 5 metabolites remained unmapped to any significant pathway.

The most striking finding was the enrichment of choline metabolism in cancer (85.6-fold enrichment, FDR = 0.0008), which exhibited the strongest signal in the entire dataset. This pathway is critically important in cancer biology, as malignant cells demonstrate increased choline kinase activity and elevated phosphocholine levels. The involvement of phosphorylcholine and choline in this pathway suggests potential alterations in cell membrane biosynthesis and signaling cascades characteristic of transformed cells. The second most enriched pathway was carbohydrate digestion and absorption (52.3-fold enrichment, FDR = 0.0003), involving galactose, fructose, and butyric acid. This enrichment indicates significant alterations in carbohydrate metabolism and absorption processes, potentially reflecting dysregulation in glucose homeostasis, intestinal absorption capacity, or gut microbiome-mediated metabolic activities. Butyric acid, a short-chain fatty acid produced by intestinal bacteria, plays crucial roles in colonocyte energy metabolism and maintenance of intestinal barrier function. The third most enriched pathway was glycine, serine and threonine metabolism (29.4-fold enrichment, FDR = 0.0008), which is central to one-carbon metabolism supporting critical cellular processes including nucleotide synthesis, methylation reactions, and redox homeostasis. This enrichment suggests alterations in amino acid metabolism that could affect cellular proliferation, epigenetic regulation, and antioxidant defense mechanisms.

Overall, the enrichment pattern reveals coordinated alterations across multiple metabolic domains, including cancer-related choline metabolism, carbohydrate processing, amino acid metabolism, and various transport systems. This multi-pathway involvement suggests systemic metabolic reprogramming rather than isolated pathway dysregulation. The detailed results of the pathway enrichment analysis are presented in the following table, with color coding to indicate enrichment levels: very high enrichment (greater than 50-fold) shown with a light red background, high enrichment (20–50-fold) with a light yellow background, and moderate enrichment (less than 20-fold) with a white background.

## 4. Discussion

In this exploratory metabolomics study, we examined metabolic changes in long-livers and observed distinct metabolic patterns that may differentiate individuals with metabolic perturbations from those with preserved metabolic stability. Our results suggest differences in metabolite composition, pathway enrichment, and clinical associations within the cohort, providing preliminary insights into potential mechanisms of metabolic adaptation during extreme aging.

Our metabolomics analysis revealed two distinct metabolic patterns in long-livers, suggesting that despite reaching extreme age, some individuals undergo metabolic shifts that may reflect underlying physiological and pathological processes. The observation of significantly altered metabolites in the metabolically altered group provides preliminary support for this notion. The upregulation of butyric acid derivatives, D-galactose, D-glucose, L-methionine, choline, and phosphorylcholine may indicate potential disruptions in amino acid metabolism, carbohydrate metabolism, and lipid processing. Notably, butyric acid derivatives were absent in the metabolically stable group, suggesting that short-chain fatty acid metabolism may play a role in long-livers exhibiting metabolic alterations. Given that butyrate and its derivatives are gut microbiota-derived metabolites known to lower arterial blood pressure [22], their presence in the metabolically altered group may have had a protective effect in individuals with cardiovascular conditions, although this hypothesis requires validation in larger cohorts.

These findings are consistent with our previous gut microbiota study of long-livers, which revealed that in this group of people individuals possessed microbiota with significantly higher potential to produce butyrate compared to healthy middle-aged adults. In that study, we observed that centenarian microbiota was characterized by elevated butyrate-producing capacity (*p* = 0.016, MaAsLin), which we hypothesized may protect long-livers from low-grade inflammation and subsequent metabolic disorders [23]. The current metabolomics data provides preliminary support for this hypothesis by suggesting elevated butyric acid derivatives in a subgroup of long-livers with metabolic alterations, which may indicate an active protective mechanism mediated by gut microbiota-derived metabolites, though causality cannot be inferred from this cross-sectional design.

Our clinical data analysis further revealed that long-livers with metabolic alterations, characterized by elevated butyrate levels, formed a distinct clinical subgroup that appeared to exhibit a higher prevalence of cardiovascular diseases, including coronary artery stenosis and atherosclerosis. Given the aforementioned role of butyrate in lowering arterial blood pressure, its elevated levels in this subgroup may have contributed to their exceptional longevity despite the presence of cardiovascular pathologies, although this remains speculative. This observation is consistent with our previous finding that long-livers were “unexpectedly healthy” despite their extreme age, suggesting that protective mechanisms such as enhanced butyrate production may counterbalance age-related pathological processes.

The integration of our current metabolomics findings with previous microbiota data provides preliminary evidence for a potential gut-metabolome axis in exceptional longevity. The presence of anti-inflammatory Bifidobacterium genus and enhanced butyrate-producing capacity in centenarian microbiota, coupled with the current observation of elevated butyric acid derivatives in metabolically altered long-livers, may suggest a coordinated response to age-related metabolic stress. This protective mechanism may help explain how some individuals can achieve extreme longevity while maintaining cardiovascular health or surviving despite cardiovascular pathologies, although longitudinal studies are needed to establish temporal relationships.

However, several important confounding factors must be considered when interpreting these findings. Medications commonly used by long-livers, including statins, metformin, NSAIDs, and antibiotics, may significantly influence metabolite profiles. Dietary factors, particularly protein and fiber intake, are known modulators of both gut microbiota composition and metabolite production. Furthermore, renal function, which was significantly impaired in the metabolically altered group (CKD *p* = 0.002), may affect metabolite clearance and accumulation. The observed metabolic differences may also reflect underlying inflammatory status or other age-related comorbidities rather than representing primary metabolic alterations. Without controlling for these variables, the independent contribution of gut microbiota-derived metabolites to the observed patterns remains uncertain.

Overall, these hypothesis-generating findings highlight the heterogeneity of metabolic aging in long-livers and provide preliminary insights into biochemical pathways that may be associated with longevity. While some long-livers appear to maintain metabolic stability, others show significant changes associated with distinct clinical profiles and potential gut microbiota-mediated protective mechanisms. Future research should examine the causal links between these metabolic changes, gut microbiota composition, and age-related diseases to determine whether specific metabolic signatures contribute to longevity or simply reflect underlying pathophysiological processes. Understanding these mechanisms may open new avenues for targeted interventions, potentially including microbiota-based therapies, to promote healthy aging and reduce the burden of metabolic diseases in older adults.

### Study Limitations

This study has several important limitations that should be considered when interpreting the results.

**Sample Size**. The small sample size of the metabolically altered group (n = 6) is a significant limitation. Although our post hoc power analysis indicated 100% statistical power for detecting very large effect sizes (Cohen’s d > 2.0) and 99.9% power for L-serine specifically, the study was underpowered to detect moderate or small metabolic differences. The stringent FDR threshold (FDR < 0.01) minimizes false positive findings, with an expected <0.65 false positives among 15 significant metabolites, but limits our ability to detect subtle metabolic alterations that may be biologically relevant. These findings should therefore be considered exploratory and require validation in larger independent cohorts.

**Cross-Sectional Design**. The cross-sectional nature of this study prevents inference of causality or temporal relationships between metabolic alterations and clinical outcomes. Reverse causation remains possible—metabolic changes may result from rather than contribute to cardiovascular disease or extreme longevity. Longitudinal studies tracking metabolic trajectories over time are needed to establish whether observed metabolic patterns precede, accompany, or follow the development of age-related conditions.

**Group Assignment**. The classification of long-livers into metabolically altered and stable groups was based on visual inspection of PCA plots, introducing subjectivity into the analysis. Although group assignment was performed prior to statistical testing to avoid circular analysis, future studies should employ objective clustering algorithms with predefined criteria. The choice of 3 standard deviations as a threshold for cluster separation, while statistically defensible, was arbitrary and may not reflect true biological boundaries.

**Uncontrolled Confounders**. Several potential confounding variables were not systematically controlled in this analysis. Medication use, including statins, metformin, NSAIDs, and antibiotics, may substantially alter metabolite profiles independently of underlying metabolic state. Dietary factors, particularly protein and fiber intake, directly influence gut microbiota composition and metabolite production but were not quantified. Renal function, which differed significantly between groups (CKD *p* = 0.002), may affect metabolite clearance and accumulation patterns. Furthermore, inflammatory status and other unmeasured comorbidities may confound observed associations. Without adjusting for these variables, the independent contribution of metabolic alterations to clinical outcomes remains uncertain.

**Metabolomics Methodology**. The untargeted metabolomics approach used in this study provides semi-quantitative rather than absolute metabolite concentrations. Metabolite identification reached MSI Level 2 (putative annotation based on spectral and physicochemical similarity), which carries inherent uncertainty compared to confirmation with authentic standards. Targeted metabolomics with validated quantification methods is needed to confirm the identity and absolute concentrations of key metabolites, particularly butyric acid derivatives.

**Generalizability**. This study was conducted in a single geographic region (Russia) with specific population characteristics. The metabolic patterns observed in this cohort may not extrapolate to long-livers from other ethnicities, geographic regions, or with different environmental exposures. Cultural factors influencing diet, lifestyle, and healthcare access may substantially modify the relationship between metabolic patterns and longevity.

**Statistical Conservatism**. While our stringent statistical approach (FDR < 0.01) minimizes false positive findings, it may have resulted in overlooking metabolites with moderate effect sizes that could be biologically meaningful. The trade-off between Type I and Type II error rates is inherent to exploratory studies with small samples, and some true metabolic differences may have been missed due to limited statistical power.

Despite these limitations, the strong effect sizes observed (particularly for L-serine and butyric acid derivatives), the stringent statistical thresholds applied, and the consistency with previous microbiota findings provide confidence that the identified metabolic patterns reflect genuine biological differences warranting further investigation. Future studies with larger sample sizes, longitudinal designs, controlled confounders, and validation in independent cohorts are essential to confirm these exploratory findings and establish their clinical relevance.

## 5. Conclusions

This study demonstrates significant metabolic heterogeneity among long-livers, identifying a distinct subgroup characterized by elevated butyric acid metabolism and associated cardiovascular pathologies. Through integrated metabolomics and clinical data analysis, we revealed that approximately 11% of long-livers exhibit metabolic alterations primarily involving butyrate derivatives, amino acid metabolism, and carbohydrate processing pathways. Importantly, this metabolically altered group showed a higher prevalence of cardiovascular diseases, including coronary artery stenosis and atherosclerosis, yet achieved exceptional longevity, suggesting the presence of protective compensatory mechanisms.

Our findings support the hypothesis that gut microbiota-derived butyrate may serve as a protective factor in exceptional aging, potentially counterbalancing cardiovascular pathologies through anti-inflammatory and blood pressure-lowering effects. The absence of butyric acid derivatives in the metabolically stable group and their exclusive presence in the cardiovascular disease-associated subgroup highlight the complex relationship between metabolic adaptation, disease survival, and longevity. These results contribute to our understanding of the biochemical basis of extreme aging and suggest that metabolic signatures in long-livers reflect not merely healthy aging but also successful adaptation to age-related pathological processes.

Future investigations should focus on establishing causal relationships between specific metabolic pathways, gut microbiota composition, and longevity outcomes to develop targeted interventions for promoting healthy aging. The identification of butyrate metabolism as a potential therapeutic target opens new avenues for microbiota-based therapies aimed at enhancing metabolic resilience and cardiovascular protection in aging populations.

## Figures and Tables

**Table 1:** Baseline Characteristics of Study Population (n = 53).

Characteristic	Total (n = 53)	Metabolic Alterations (n = 6)	No Metabolic Alterations (n = 47)	*p*-Value
Demographics				
**Age, years**	98.0 [97.0–99.0]	97.5 [97.0–98.0]	98.0 [97.0–99.0]	0.26
**Male sex, n (%)**	7 (13.2)	2 (33.3)	5 (10.6)	0.17
**BMI, kg/m^2^**	24.6 [21.8–27.6]	25.4 [21.1–27.4]	24.6 [22.0–27.7]	0.79
**Centenarians (age ≥ 100 years), n (%)**	8 (15.1)	0 (0.0)	8 (17.0)	0.57
**Functional and Social Status**				
**Disability, n (%)**	46 (86.8)	6 (100.0)	40 (85.1)	0.58
Comorbidities				
**Hypertension, n (%)**	42 (79.2)	5 (83.3)	37 (78.7)	1.00
**Coronary artery disease, n (%)**	32 (60.4)	5 (83.3)	27 (57.4)	0.38
**Chronic heart failure, n (%)**	19 (35.8)	4 (66.7)	15 (31.9)	0.17
**Atrial fibrillation, n (%)**	4 (7.5)	0 (0.0)	4 (8.5)	1.00
**Diabetes mellitus, n (%)**	1 (1.9)	0 (0.0)	1 (2.2)	1.00
**COPD, n (%)**	7 (13.2)	1 (16.7)	6 (12.8)	1.00
Laboratory Parameters				
**Hemoglobin, g/L**	123.0 [112.5–130.5]	129.5 [120.8–131.5]	122.0 [112.0–130.0]	0.30
**Leukocytes, ×10^9^/L**	6.4 [5.2–7.8]	6.3 [5.9–6.5]	6.6 [5.1–7.8]	0.94
**Platelets, ×10^9^/L**	233.0 [180.5–261.5]	163.0 [140.2–228.5]	234.0 [187.0–269.0]	0.07
**Albumin, g/L**	38.3 [35.9–40.1]	39.0 [38.1–39.6]	38.1 [35.3–40.5]	0.73
**Creatinine, µmol/L**	92.3 [79.4–106.4]	96.3 [88.1–124.4]	92.3 [78.9–105.2]	0.39
**AST, U/L**	20.1 [17.5–25.2]	23.4 [19.5–27.3]	19.4 [17.1–25.0]	0.19
**ALT, U/L**	8.9 [7.5–12.1]	12.4 [9.8–18.0]	8.7 [7.2–11.7]	0.06

Data presented as median [IQR], or n (%). *p*-values calculated using Mann–Whitney U test for continuous variables and Fisher’s exact test for categorical variables. BMI, body mass index; COPD, chronic obstructive pulmonary disease; CKD, chronic kidney disease; AST, aspartate aminotransferase; ALT, alanine aminotransferase.

**Table 2:** Differentially Abundant Metabolites Between Groups.

Rank	Metabolite	Log2(Fold Change)	*p*-Value	Q-Value	Metabolic Alterations (log2)	No Metabolic Alterations (log2)
1	D-Galactose	6.86	1.09 × 10^−7^	8.87 × 10^−7^	7.64	0.78
2	2-Hydroxy-3-methylbutyric acid	6.77	7.97 × 10^−8^	8.63 × 10^−7^	7.54	0.77
3	L-Alpha-aminobutyric acid	6.51	8.58 × 10^−5^	4.65 × 10^−4^	7.25	0.74
4	D-Fructose	6.49	9.77 × 10^−8^	8.87 × 10^−7^	7.23	0.73
5	Ethanol	6.29	6.12 × 10^−4^	1.63 × 10^−3^	6.99	0.70
6	Phosphorylcholine	6.29	8.60 × 10^−6^	6.51 × 10^−5^	6.98	0.69
7	Butyric acid	6.24	1.51 × 10^−5^	9.79 × 10^−5^	6.93	0.69
8	Choline	6.11	3.62 × 10^−8^	5.45 × 10^−7^	6.79	0.69
9	2-Ketobutyric acid	5.81	3.88 × 10^−3^	8.39 × 10^−3^	6.46	0.65
10	Trimethylamine N-oxide	5.75	1.89 × 10^−9^	4.09 × 10^−8^	6.40	0.65
11	2-Hydroxybutyric acid	5.75	1.30 × 10^−3^	2.81 × 10^−3^	6.39	0.64
12	Alpha-Hydroxyisobutyric acid	5.10	1.42 × 10^−9^	4.09 × 10^−8^	5.67	0.58
13	3-Hydroxybutyric acid	4.84	4.19 × 10^−8^	5.45 × 10^−7^	5.38	0.55
14	Ketoleucine	4.83	1.00 × 10^−4^	4.87 × 10^−4^	5.37	0.54

Welch’s *t*-test with Benjamini–Hochberg FDR correction applied. Log2(Fold Change) calculated as difference in means in log2-transformed space. Data normalized using log2(x + 1) transformation. Significance thresholds: q-value < 0.01, |log2FC| > 2. L-Serine (rank 1) was previously missing from published results due to data processing error.

**Table 3:** Pathway enrichment analysis.

№	Pathway ID	Pathway Name	Category	Metabolites	Fold Enrichment (95% CI)	*p*-Value	FDR	Sig.	Metabolite IDs
**1**	map05231	Choline metabolism in cancer	Human Diseases	2	85.6 (72.8–98.4)	2.28 × 10^−4^	0.0008	***	C00588/C00114
**2**	map00552	Teichoic acid biosynthesis	Metabolism	2	78.5 (66.2–90.7)	2.73 × 10^−4^	0.0008	***	C00588/C00114
**3**	map02030	Bacterial chemotaxis	Cellular Processes	1	78.5 (61.1–95.8)	1.27 × 10^−2^	0.0127	*	C00124
**4**	map04973	Carbohydrate digestion and absorption	Organismal Systems	3	52.3 (44.2–60.5)	2.17 × 10^−5^	0.0003	***	C00124/C00095/C00246
**5**	map00670	One carbon pool by folate	Metabolism	2	36.2 (27.9–44.5)	1.32 × 10^−3^	0.0028	**	C00065/C00114
**6**	map04978	Mineral absorption	Organismal Systems	2	32.5 (24.6–40.3)	1.65 × 10^−3^	0.0031	**	C00065/C00124
**7**	map00260	Glycine, serine and threonine metabolism	Metabolism	3	29.4 (23.3–35.5)	1.25 × 10^−4^	0.0008	***	C00065/C00114/C00109
**8**	map00564	Glycerophospholipid metabolism	Metabolism	3	25.2 (19.6–30.9)	1.98 × 10^−4^	0.0008	***	C00065/C00588/C00114
**9**	map00640	Propanoate metabolism	Metabolism	2	22.4 (15.9–29.0)	3.44 × 10^−3^	0.0049	**	C00109/C05984
**10**	map00270	Cysteine and methionine metabolism	Metabolism	3	20.8 (15.6–25.9)	3.52 × 10^−4^	0.0009	***	C00065/C02356/C00109
**11**	map00052	Galactose metabolism	Metabolism	2	20.5 (14.2–26.7)	4.11 × 10^−3^	0.0049	**	C00124/C00095
**12**	map00650	Butanoate metabolism	Metabolism	2	20.0 (13.9–26.2)	4.29 × 10^−3^	0.0049	**	C00246/C01089
**13**	map04974	Protein digestion and absorption	Organismal Systems	2	20.0 (13.9–26.2)	4.29 × 10^−3^	0.0049	**	C00065/C00246
**14**	map02060	Phosphotransferase system (PTS)	Environmental Information Processing	2	16.5 (10.9–22.1)	6.25 × 10^−3^	0.0067	**	C00124/C00095
**15**	map02010	ABC transporters	Environmental Information Processing	3	10.2 (6.7–13.8)	2.76 × 10^−3^	0.0046	**	C00065/C00095/C00114

Significance levels are denoted as follows: * *p* < 0.05, ** *p* < 0.01, and *** *p* < 0.001, all after FDR correction. Four metabolites—TMAO, linoleic acid, 2-hydroxy-3-methylbutyric acid, and ethanol—were not significantly enriched in any pathway at FDR < 0.05 and are therefore classified as unmapped metabolites. The table uses color coding to indicate enrichment levels: very high enrichment (>50-fold) is shown with a light red background, high enrichment (20–50-fold) with a light yellow background, and moderate enrichment (<20-fold) with a white background.

## Data Availability

The data that support the findings of this study are available from the corresponding author upon reasonable request.
